# Snacking Consumption among Adults in the United States: A Scoping Review

**DOI:** 10.3390/nu15071596

**Published:** 2023-03-25

**Authors:** Jean Pierre Enriquez, Elizabeth Gollub

**Affiliations:** School of Nutrition and Food Sciences, Louisiana State University Agricultural Center, Baton Rouge, LA 70803, USA; jenriq5@lsu.edu

**Keywords:** eating occasion, food consumption, snacking, snack selection, snack frequency

## Abstract

Snacks are a staple of the American diet, contributing to approximately 20% of energy intake. Most U.S. adults consume one to three snacks/day, yet few reviews have focused on snacking among this population. This scoping review was conducted to characterize snacks and snacking occasions among U.S. adults to further inform healthy eating practices. The protocol was prepared following the PRISMA-Extension for Scoping Reviews. Three web databases were used to identify articles using snacking or eating occasions as primary or secondary outcomes among U.S. adults. A search strategy was developed using subject headings, truncation, and phrase searching in the title and abstract of articles published between 2010 and 2022. A two-stage, multi-step screening process identified 31 of 4795 publications as meeting the inclusion criteria. Findings included identification of snacking themes, e.g., cues and motivations; diet composition; and weight management. Food quality, time of consumption, and convenience emerged as characteristics of snacking; time of day was found to influence food choice. Snacks contribute to or detract from a healthy diet. Strategic selection of healthy snack options can improve diet quality. Applied to nutrition education, this information can help strengthen programs or policies, ultimately contributing to health and wellbeing.

## 1. Introduction

Snacks are a staple of the American diet, accounting for nearly a quarter (22%) of total energy intake among adults [1,2]. Currently, more than 90% of U.S. adults report eating one or more snacks on any given day [1], with an average of between 1.2 and 3.0 snacking occurrences per day [3]. Over the past few decades, snacking, which may be a response to physiological or emotional “hunger” for energy and/or satiety [4], has been increasing in frequency and volume [5].

Snacking, like any dietary behavior, can be practiced in a manner that is healthful or not [6]; therefore, it can be associated with both positive and negative outcomes. For example, several researchers have reported that snacking may help individuals achieve recommended intakes of fruits, dairy products, vitamins, minerals, and fiber, and that it can help individuals avoid digestive and metabolic overload triggered by fewer, larger meals [7,8,9,10]. On the other hand, as reported by Skoczek-Rubiska and Bajerska [11], consumption of snacks beyond energy needs may contribute to obesity. Unhealthy snacking patterns and routine consumption of unhealthy snack foods diminish overall diet quality. Poor diet is a well-established risk factor for noncommunicable diseases, which are the leading causes of death worldwide [12]. Nutrient rich or otherwise, snacking has become an influential component of the daily diet [13].

Context is a key determinant of food selection, eating, and food consumption behaviors. Adults snack in a variety of settings and times. For example, they might snack at home or at work, alone or with others, late at night in front of the television, as part of social functions, while commuting, or while on the computer [11]. Any food can be consumed as a snack. However, snack foods are often easy-to-eat products, such as cookies and potato chips, that tend to have a poor nutrient profile, i.e., low in micronutrients, high in sugars and/or added fat, and low in fiber [14]. Regardless of whether it is fried or fresh, or consumed in the morning or at midnight, in all cases, food ingested while snacking, as with meals, influences nutrient and energy balance [15,16].

Any time a person eats or drinks is referred to as an eating occasion [17]. Relative to meals, snacking can be irregular in terms of schedule and composition [11]. Meals are usually defined as structured eating occasions that take place between the hours of 6:00 and 10:00 a.m., 12:00 and 3:00 p.m., and 6:00 and 9:00 p.m., corresponding to breakfast, lunch, and dinner, respectively. Eating occasions outside of these times, typically characterized by more casual consumption of small quantities of food, are generally classified as snacks [18,19].

Daily food selection can be a complicated process driven by a variety of elements [20]. Although taste, price, healthfulness, and convenience are major drivers of food choice [21], social norms have a significant impact on food consumption patterns, in terms of both choice and quantity [22]. For example, people driven by health tend to make healthier food selections, regardless of convenience; however, when socially pressured by those making less healthy choices, they too might consume more popular snack foods [23]. In general, people eat significantly more with others than when they are alone, a phenomenon known as social facilitation of eating [24]. These social influences of eating behaviors are considered ‘normal’ [4] and tend to operate in tandem with physiological variables such as hunger [25].

That people eat at all stems from hunger, sensory appeal, and social conventions that affect modulation and automaticity [26,27]. Among adults, the primary positive motivations for eating have been reported as health, hunger, pleasure, sociability, and tradition [23], with need and hunger, pricing, habit, sociability, traditional eating [26], and the nature of food influencing specific food choice [28]. However, most of the research in this field related to snacking has focused on how various eating behaviors affect energy balance and weight status [29,30].

To date, evidence from scoping, systematic, or traditional literature reviews on the topic of snacking has been focused on children or adolescent populations [31,32,33,34,35,36,37], or on universal snacking consumption recommendations [38,39]. For the most part, the literature has reported on snacking in terms of food choice and quantities, in relation to overall food consumption and weight status or management. In fact, “nutrient-dense snacks” are included in the current U.S. Dietary Guidelines as a means of, for example, increasing critical nutrients and moderating appetite and/or blood sugar, to help promote and maintain healthy weight, and reduce risk to chronic disease across U.S. populations [40]. Diet-related chronic disease affects approximately 60% of U.S. adults [41]. However, little is known about the characterization, quantitative or qualitative, of snack consumption, a significant dietary component, among U.S. adults in particular. This scoping review aims to contribute to this gap by asking “among U.S. adults, what is being consumed as snacks, what motivates snack food choice, and when does snacking occur?” This work intends to present findings that could inform recommendations and nutrition education practices to further support healthy eating.

## 2. Methods

### 2.1. Protocol and Registration

The protocol was prepared following the Preferred Reporting Items for Systematic Reviews and Meta-Analysis Extension for Scoping Reviews (PRISMA-ScR). We first created an a priori protocol that outlined the study questions, goals, inclusion standards, and methodologies. The final version was registered in the Open Science Framework on 18 April 2022 and approved on 21 April 2022 [42].

### 2.2. Eligibility Criteria

Only publications from peer-reviewed scientific journals and those focused on adults (18 years old or over) living in the U.S. were considered for this scoping review. Adults were defined in accordance with the human age categories from Nithyashri and Kulanthaivel [43]. Included articles were those published from January 2010 to May 2022, written in English or Spanish, because one reviewer (JPE) is bilingual. Quantitative, qualitative, or mixed-method approaches were included to allow for more extensive navigation among snacking patterns in terms of perceptions and rates, obtained from studies using a variety of survey instruments.

Articles were excluded if the research did not describe health status by food consumption, snacking and/or meal frequency, determinants of healthy aging, drivers of snacks and snacking, exposure to relatively unknown foods, or ready-to-eat foods. Furthermore, papers were excluded if the research was not conducted in the U.S.

### 2.3. Information Sources

Pubmed, Ovid, and Scopus databases were used to identify potential works for inclusion in this review. Database filters were used to select for desired dates (January 2010 to May 2022). Consultation with the university librarian helped inform the research strategy, which was developed by JPE, the first author, which was then discussed and enhanced with EG, the second author. Database research was carried out from 10 May to 26 May 2022, using subject headings, truncation, and phrase searching in the title and abstract fields. All papers that met the inclusion criteria received an initial screening.

### 2.4. Search

A pilot search was made on PubMed as a means of reviewing relevant findings for additional key words and index phrases. This helped reduce the number of irrelevant returns. The research strategy refined through the PubMed pilot is presented in Table 1. The same strategy was also used with Ovid and Scopus databases.

### 2.5. Selection of Source of Evidence

**Figure 1:**
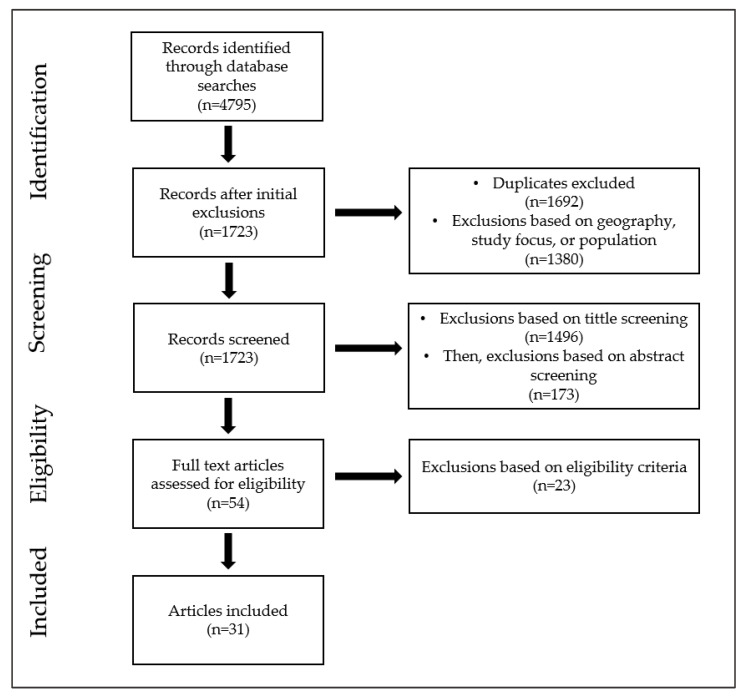
Flow diagram of literature search and study selection process.

All identified records (*n* = 4795) were stored in individual e-folders for each database. Duplicates (*n* = 1692) were removed by JPE, the primary researcher. This was followed by the removal of returns if they were animal, plant, or laboratory studies; studies involving non-U.S. populations or including children or adolescents; and studies that did not involve human nutrition, public health, or foods (Figure 1). From this cleaned set of returns (*n* = 1723), the scoping review then proceeded as a two-stage screening process. Stage 1, a preliminary stage, weeded out articles based on title. Using the “Random” function on Excel, 345 records (20%) were selected for review by JPE, as well as DQ, a research assistant. Each title was screened against the inclusion criteria to exclude those that were irrelevant, i.e., articles in other fields. Screening agreement between JPE and DQ was very strong (83%); therefore, JPE completed the tittle screening independently. Stage two, the abstract screening, was conducted by the two reviewers. JPE screened all abstracts against the inclusion criteria, then DQ repeated the screening process, confirming or rejecting the records. From the abstract review, 54 articles were retained for comprehensive, full-text screening, from which 31 articles met the inclusion criteria. Discrepancies were handled by discussion to reach consensus after a full-text screening.

### 2.6. Data Collection

JPE collected data from the group of included studies. Author, publication year, location, objectives, study population and sample size, methods, and major findings were extracted for transfer into a data table. Another reviewer, EG, examined the information extracted to provide feedback, resolve potential discrepancies/incongruities, and enhance clarity.

## 3. Results

### 3.1. Description of Study Findings

#### 3.1.1. Reach of Included Studies

The location of the studies covered by this review ranged from local to national. As shown in Table 2, twelve studies were conducted in a single town or city [44,45,46,47,48,49,50,51,52,53,54,55]. Four studies were conducted in a single state [48,56,57,58], four were multistate studies [59,60,61,62], one involved an entire geographic region [63], and six were nationwide [3,10,64,65,66,67]. Four studies that were “conducted in the U.S.” provided no specific location information [68,69,70,71]. Where possible, we sub-grouped these studies by region to help detect any differences. The Northeast region hosted seven studies, followed by Southeast and West with five studies each; the Midwest had four and the Southwest had two. One study reported participants from each of the five regions but did not indicate it was a nationwide study [62]. However, no patterns or conclusions could by drawn based on this classification.

Study sizes ranged from 4 to 19,427 participants. Eighteen studies reported 602 or less participants [44,47,48,49,50,51,52,53,54,56,57,58,60,61,62,63,71], while ten studies reported more than 1000 [3,10,44,45,46,64,65,66,69,70]. Three of the studies did not report sample size [55,67,68]. The smaller studies (602 or less participants) generally identified actions that characterized eating habits and motivations, planned or spontaneous, linked to snacking behaviors among the participants. The larger studies, i.e., those with more than 1000 participants, suggested a range of recommendations, relating snacking to physical activities, health outcomes, weight concerns, and nutrient absorption. Regardless of sample size, diet quality emerged as an important factor related to snacking consumption.

Most of the studies in this review (25/31) reported on adults aged 18 years or older. However, two studies reported specifically on adults older than 60 years [10,65]. It is important to highlight that age was reported in terms of intervals and/or means. Among studies with a mean participant age of 40 years or less, the findings focused on snacking in terms of frequency per day, calorie consumption, and weight or obesity status. In these studies, opportunities for educational interventions and health promotion efforts were highlighted. Among studies with a mean participant age of 40 years or more, findings could be categorized as snacking in terms of food type, consumption of leftovers, and health status.

Though participants’ sex/gender, race, or ethnicity could not be effectively quantified from the studies included in this scoping review, we were able to categorize participant target populations for approximately half of the studies. These included workers [44,48,54,56,57], people with obesity [51,53,60], university students [47,48,55,63,71], and the general population [10,45,46,58,64,69,70]. Twelve out of thirty-one studies did not report this information [3,44,48,49,50,52,61,62,65,66,67,68]. Still, when reviewed in conjunction with age ranges and geographical locations, this vocation or status sketch helped indicate that these studies involved reasonably diverse participant samples.

#### 3.1.2. Study Design

A variety of research designs were used within the group of studies included in this review. Of the thirty-one, fifteen were cross-sectional studies, seven were randomized controlled trials, two were prospective studies, one was a factorial cross, one was a longitudinal pilot, and one was a case study (Table 2); each contributed to the evaluation of the relationships among foods, snacks, consumption behaviors, body weight, and health. Given the enormous impact of diet on health and given that everyone eats (and most people snack), the studies included in our review provide valuable, actionable information.

A variety of data collection methods were used among the included studies. Twenty studies collected data through questionaries or surveys [3,10,44,47,48,52,54,57,58,60,62,63,65,66,67,68,69,70]; seven conducted interviews [45,46,53,56,61,64,71]; three utilized smartphone apps [49,51,61]; one recorded self-checklist reports [48]; and one received data from a food purchase database [55]. Reviewing these methods in terms of findings helped solidify our understanding of the utility of each of these tools/approaches.

#### 3.1.3. Study Outcome Measures

Table 3 summarizes outcome measures from the 31 studies. Half of the studies (15/31) collected data to characterize consumption. Of these, eight studies [3,20,45,48,52,60,64,68] included both meal and snacking occasions, while four studies [44,65,67,72] were exclusively related to snack consumption. One study included the peak time of food consumption [58], while three studies included quality and utility of snacks [51,55,62]. Taken together, these studies highlighted the influence of timing on snacking or eating occasions and the quality or composition of foods/snacks.

**Table 2:** Description of included studies.

Author	Year	Sample Size	Years	Design	Methods	Population	Location	U.S. Regions
Barrington and Beresford [44]	2019	2265	$\bar{x}$ = 43	Randomized Controlled Trial	Questionnaire	Workers (manufacturing, transportation, utilities, household, and others)	Seattle, Washington	West
Berryman et al. [66]	2021	10,112	≥19	Cross-sectional	Survey	NR	Nationwide	Nationwide
Close et al. [57]	2016	388	$\bar{x}$ = 42.4	Cross-sectional	Questionnaire	Health insurer workers	North Carolina	Southeast
Cowan et al. [3]	2020	9633	$\bar{x}$ = 48.3	Cross-sectional	Survey	NR	Nationwide	Nationwide
Ebel and Byker [56]	2022	4		Case study	Semi-structured Interview	Store managers	Montana	West
Grimes et al. [45]	2018	1834	32–70, $\bar{x}$ = 53.2	Prospective	Interview	African American and White adults	Baltimore, Maryland	Northeast
Hess et al. [69]	2017	$\bar{x}$ = 5000	NR	Cross-sectional	Survey	Families (household leaders)	U.S., non-specified	U.S., non-specified
House et al. [47]	2018	92	$\bar{x}$ = 18.8	Cross-sectional	Survey	Hispanic college freshmen	Austin, Texas	Southwest
Kong et al. [53]	2011	123	$\bar{x}$ = 58	Randomized Controlled Trial	Interview	Postmenopausal overweight-to-obese women	Seattle, Washington	West
Kuczmarski et al. [46]	2017	7177	30 to 64, $\bar{x}$ = 47	Factorial Cross	Interview	African American and White adults	Baltimore, Maryland	Northeast
Laska et al. [52]	2011	48	18 to 23	Cross-sectional	Survey	NR	Minneapolis, Minnesota	Midwest
Liu et al. [62]	2015	226	18 to 85, $\bar{x}$ = 40	Cross-sectional	Questionnaire	NR	Los Angeles, California; Chapel Hill, North Carolina; Columbus, Ohio; Philadelphia, Pennsylvania; and Albuquerque, New Mexico.	All regions
Malaeb et al. [51]	2020	20	$\bar{x}$ = 45.5	Randomized Controlled Trial	Smartphone App: survey	Metabolically healthy, overweight, or obese adults	Minneapolis, Minnesota	Midwest
McCurley et al. [54]	2022	602	$\bar{x}$ = 43.6	Cross-sectional	Survey	Hospital employees	Boston, Massachusetts	Northeast
Mills et al. [70]	2011	1099	$\bar{x}$ = 49.6	Cross-sectional	Questionnaire	Midlife women	Nine metropolitan and micropolitan statistical areas	U.S., non-specified
Murakami and Livingstone [67]	2015	NR	20 to ≥60	Cross-sectional	Survey	NR	Nationwide	Nationwide
Murakami and Livingstone [64]	2016	19,427	≥20	Cross-sectional	Face to face or phone interview	General population, excluding pregnant and lactating	Nationwide	Nationwide
Perrigue et al. [50]	2016	12	18 to 50	Randomized Controlled Trial	Self-Report	NR	Seattle, Washington	West
Phan and Chambers [20]	2016	198	18 to 74	Pilot study	Survey	University faculty, staff, and students	Kansas	Midwest
Phan and Chambers [48]	2018	100	18 to 74	Cross-sectional	Survey	NR	Manhattan, Kansas	Midwest
Popp et al. [59]	2021	85	$\bar{x}$ = 56	Cross-sectional	Smartphone App; face to face or phone interview	NR	New York	Northeast
Reid et al. [68]	2014	NR	$\bar{x}$ = 31.7	Cross-sectional	Questionnaire	NR	U.S., non-specified	U.S., non-specified
Roe et al. [49]	2020	18	$\bar{x}$ = 50.8	Cross-sectional	Smartphone App: survey	NR	Baton Rouge, Louisiana	Southeast
Schwedhelm et al. [58]	2022	420	NR	Prospective	Questionnaire	Pregnant or postpartum females	North Carolina	Southeast
Shimizu et al. [63]	2010	122	NR	Randomized Controlled Trial	Survey	Undergraduate University Students	Northeast	Northeast
Taetzsch et al. [60]	2021	229	$\bar{x}$ = 40.9	Cross-sectional	Questionnaire	Overweight or obese female dependent of active duty or retired military personnel	Massachusetts, Connecticut, New York, Colorado, and Kentucky	Northeast, West, Southeast
Thomas et al. [59]	2013	490		Cross-sectional	Online survey	NR	Arkansas, Florida, Georgia, Mississippi, North Carolina, Tennessee, Virginia.	Southeast
Wansink et al. [71]	2010	122	19 to 25	Cross-sectional	Interview	College students	U.S., non-specified	U.S., non-specified
Wansink et al. [55]	2013	NR	NR	Longitudinal	Food Purchase Database	University Students	Ithaca, New York	Northeast
Xu et al. [65]	2013	2333	≥60	Cross-sectional	Survey	NR	Nationwide	Nationwide
Zizza et al. [10]	2010	2056	≥65	Cross-sectional	Survey	Older adults	Nationwide	Nationwide

**Table 3:** Eating occasions, timeframes, and key outcomes of included studies.

Author(s)	Type of Eating Occasion; Timeframe	Study Highlights (Outcome Variables, Results, and/or Background)
Barrington and Beresford [44]	Snacks: Morning: 12:00 a.m. to 11:00 a.m. Mid-day: 11:00 a.m. to 4:30 p.m. Evening: 4:30 p.m. to 12:00 a.m.	Intake consisted of about 2 main meals, 1 light meal, 1.5 snacks, and 1 drink per day. Morning snacking was associated with increased fruit and vegetable consumption, while evening snacking was associated with higher BMI, higher obesogenic dietary index, and higher percent time eating while distracted. Associations with mid-day snacking were mixed.
Berryman et al. [66]	NR	Protein intake ranged from 4.9 to 16.5 g/day for combined daily snacking occasions. A greater protein consumption during combined snacking occasions was associated with decreased diastolic and systolic blood pressure and cardiovascular disease risk score, and increased HDL-Cholesterol concentrations.
Close et al. [57]	NR	Less healthful eating habits commonly included ≥2 servings/day of refined grain bread and sweet baked goods and candy. Higher frequencies of eating at fast food restaurants are associated with increased odds of eating behaviors that are less healthful independent of demographic characteristics.
Cowan et al. [3]	Meals and/or snacks: Breakfast, lunch, dinner, supper, brunch, snack, drink.	The number of daily snacking occasions varied in magnitude by the snack definition employed and generally ranged between 1.2 and 3.0 snacks per day. The frequency of snack consumption was highest when a snack was defined as any eating occasion outside of a typical mealtime (snacks + other eating between meals).
Ebel and Byker [56]	NR	Purchases of non-alcoholic beverages, dairy, and “snacks” decreased, while purchases of starchy vegetables, legumes, convenience food, and red and orange vegetables increased > 50%. Prices decreased for fruits (15.5%), “other” vegetables (10.68%), and legumes (9.81%), but increased for “snacks” (11.14%), which had the strongest purchase price.
Grimes et al. [45]	Meals and/or snacks: Breakfast, lunch, dinner, brunch, supper, snack, drink, or extended consumption.	Home breakfast consumers had significantly higher scores for all dietary components, except fatty acids and refined grains, when compared with breakfast skippers. Away-from-home consumers had higher total fruit, whole fruit, seafood and plant proteins, fatty acid, and empty calorie scores than those who skipped breakfast.
Hess et al. [69]	NR	Fruit, selected as a snack by 48% of respondents, was the most popular snack, followed by cookies (44%), chips (33%), and ice cream (33%); milk (21%) and yogurt (14%) were the least frequently consumed snacks. Yogurt, milk, and fruit were the most nutrient-dense snack categories, while ice cream, pies and cakes, and regular carbonated soft drinks were the most nutrient-poor snacks.
House et al. [47]	NR	Frequent eating was classified as averaging more than 4 eating occasions per day, while infrequent eating was classified as averaging less than 3 eating occasions per day. The average number of eating occasions per day was 3.6; the average energy consumed per eating occasion was 580.9 kcal. Infrequent eaters ate 44% less often. They consumed 27% more calories per eating occasion, but 21% fewer calories per day.
Kong et al. [53]	NR	Participants reported a mean of 6 meals/day, including 2.1 snacks/day. A total of 97% of participants reported one or more “snack meal” per day. The most common (76%) snacking period was in the afternoon (2:00 pm to 5:29 pm). Only 19% reported a mid-morning (10:30 am to 11:29 am) snack and almost 30% reported one or more snack meals after 9 pm.
Kuczmarski et al. [46]	NR	Snack consumption contributed to approximately 20% of daily energy intake. Stress and strategies to mitigate stress tended to affect energy consumption from snacks. For example, as a person tried to manage their stress, the person consumed more energy from snacks, at a rate of 5 kcal for each additional unit of stress management effort. Additionally, being male and having less education were associated with consuming more energy from snacks.
Laska et al. [52]	7:00 p.m. to 12:00 a.m. 12:00 to 5:00 a.m. 5:00 to 11:00 a.m. 11:00 a.m. to 7:00 p.m.	A large proportion of eating occasions occurred alone while watching television or engaging in other activities; were completed within a 15 min timeframe; and occurred with virtually no pre-contemplation or planning of food choices or meal selections. At-home eating occasions were associated with higher intakes of certain snacks and convenience foods (such as cookies and sweetened baked goods) and fewer traditional meal items (such as entrées, fruits, and vegetables).
Liu et al. [62]	Classification of snacks: Healthy, unhealthy, and other snack occasions.	On average, participants recorded 6.5 daily eating occasions. Snacks constituted 26% of those daily eating occasions. An average of 1.7 snaking occasions per day was reported, and classified as follows: healthy snack, 0.6; unhealthy snack, 0.8; and other snack, 0.3. The odds of consuming an unhealthy snack were 1.6 times greater for individuals with some college or vocational school compared to individuals who had completed college.
Malaeb et al. [51]	Classification of snacks: High-quality snack: nutrient-dense foods eaten individually. Low-quality snack: high-fat and/or low-nutrient foods eaten individually. Mixed-quality Snack: combination of high-quality and low-quality snack.	Compared to ad libitum food consumption, over a 12-week period, those limited to an 8 h consumption window reported fewer incomplete meals (32.9%). They reported consuming high-quality snacks 23.6% of the time and low-quality snacks 36.6% of the time. On the other hand, those who ate throughout the day reported a lower percentage (18.9%) of low-quality snacks.
McCurley et al. [54]	NR	Breakfast was the most frequently reported skipped meal; 46% of the sample reported skipping breakfast ≥ 1 day per week, whereas 36% skipped lunch and 25% skipped dinner ≥ 1 day per week. Employees who worked nonstandard shifts skipped more meals than employees who worked standard shifts. Skipping dinner ≥ 3 days per week was significantly associated with increases in systolic blood pressure.
Mills et al. [70]	NR	Snacking frequency (which averaged 2.3 times/day), breakfast consumption, and eating after 10 pm did not differ among BMI groups. Daily snacks provided 203 kcal/day (on average), less than dinner and lunch, but more than breakfast. Carbohydrate, dietary fiber, and calcium intakes increased with each additional eating occasion, whereas protein intake was significantly higher in women eating 1–3 times/day than women eating 5, 6, and ≥7 times/day.
Murakami and Livingstone [67]	NR	As meal and/or snack frequency increases, so does the likelihood of overweight or obesity. Most study participants consumed food, as a meal or snack, 3–6 times per day. These eating occasions consisted of 2–4 meals/day and 0.5–3.5 snacks/day.
Murakami and Livingstone [64]	Meals: Breakfast: about 8:00 a.m. Lunch: about 12:30 p.m. Dinner: about 8:20 p.m.	When individually considering the energy percentage, self-reporting, and time was in the range of 1.31 to 1.67 daily snacking frequency. Correlations of snack frequency with eating and energy intake were stronger than with meal frequency. In men, the highest snack frequency was observed in underweight subjects; in women, the highest snack frequency was observed in normal subjects.
Perrigue et al. [50]	NR	Based on average energy intake and frequency of morning eating (8:00 a.m. to noon), among groups of high-frequency or low-frequency eaters, peak food consumption was found to occur at 10:00 a.m. The group of high-frequency eaters also demonstrated a secondary (not as great) fullness peak during this timeframe.
Phan and Chambers [20]	Breakfast: 7:00 to 9:00 a.m. Morning snack: 8:00 to 11:00 a.m. Lunch: 11 a.m. to 1 p.m. Afternoon snack: 1:00 to 5:00 p.m. Dinner: 5:00 to 8:00 p.m. Late-night snack: 8:00 p.m. to 12:00 a.m.	Snacking is considered a personal eating event. Snacks are often consumed alone, in contrast with meal occasions. Snacks, fruits, and fruit juices were food categories consumed at snack time, regardless of time of day of snack. Nuts and seed products were preferred for mid-morning snacking, while legumes and legume products were preferred for mid-afternoon and late-night snacking, and sweets and fast foods were preferred for late-night snacks.
Phan and Chambers [48]	Meals and/or snacks: Breakfast, mid-morning snack, lunch, mid-afternoon snack, dinner, and late-night snack.	Liking a food was a stronger driver of meal consumption than snack consumption. Convenience was found to be more important for breakfast and lunch than for other eating occasions and was a secondary factor for food choice for all eating occasions. Choosing foods that reflect habits and health concerns was more common for meals than snacks. Need and hunger were core influences of food choice for all eating occasions, except late-night snacking.
Popp et al. [61]	NR	Differences in participant’s eating patterns were noted between weekdays and weekends, with greater irregularity on weekdays. This was particularly true for breakfast consumption, which seemed to coincide with activities such as watching television while being at home. People with obesity or overweight tended to be those who ate over a longer period of time, on weekdays and weekend days.
Reid et al. [68]	Meals and/or snacks: Breakfast: around 9:37 a.m. First meal (snack): around 10:02 a.m. Lunch time: around 1:32 p.m. Dinner: around 7:10 p.m. Last meal (snack): around 9:02 p.m.	The average number of daily eating occasions (meals, snacks) was 4.5. The timing of consumption of the first meal of the day (breakfast/meal 1) was not associated with total caloric intake. In contrast, eating late in day and eating closer to sleep onset was associated with a greater daily caloric intake.
Roe et al. [49]	NR	The amount of food from meals and snacks leftover by adults in their home-based settings varied by time of day and composition of the eating occasion. The percent of leftovers from snacks and meals was estimated as follows: breakfast, 12.90%; morning snack, 33.33%; lunch, 26.14%; afternoon snack, 16.67%; dinner, 28.36%; and evening snack, 0.00%. The percent of leftovers by food type was estimated as follows: vegetables and vegetable products, 19.22%; breakfast cereals, grains, and pasta, 16.25%; and meats and meat products, 16.03%.
Schwedhelm et al. [58]	Peaks of food consumption: 4:00 to 10:00 a.m. 10:00 a.m. to 2 p.m. 2:00 to 5:00 p.m. 5:00 to 8:00 p.m. 8:00 p.m. to midnight	The time windows with the highest contribution to daily energy intake were 10:00 a.m.–2:00 p.m. and 5:00–8:00 p.m., with nearly a third of the daily energy intake within each time window. An additional eating occasion was associated with an additional 161.6 kcal during pregnancy and an additional 146.4 kcal postpartum.
Shimizu et al. [63]	NR	Participants in the meal-cue condition were more likely to report that the food they ate was a meal than those in the snack-cue condition. Meal-cue participants’ actual caloric intake was significantly greater (M = 531.79) than snack-cue participants’ (M = 416.39).
Taetzsch et al. [60]	Meals and/or snacks when consuming ≥ 20 kcal.	The average daily eating interval was 11.6 h; 35.6% of the sample demonstrated a time-restricted eating pattern, 38.4% were early energy eaters, and 37.8% were bedtime eaters. Shorter daily eating intervals of 1 h, restricting daily eating to an 11 h interval, or not eating within 2 h of bedtime was associated with a decrease of 53, 140, and 235 kcal/day, respectively.
Thomas et al. [59]	NR	Four food shopping behaviors were identified: Diverse consumers (47.98%): no specific style; value-loyal consumers (16.48%): price conscious, habitual, brand and store loyal; shopping avoidance; emotional consumers (21.75%): confused by overchoice; impulsive, careless; high-conscious consumers (13.43%): perfectionists, high-quality, brand, environmental, local brand, convenience and time–energy conserving.
Wansink et al. [71]	NR	For environmental cues, eating with family is the strongest indicator of a meal, whereas standing was the strongest indicator of a snack. The profile of a snack involves eating alone for 10 min while standing, using paper plates and napkins. For food cues, low-quality food was most strongly associated with snack perceptions. The food profile of a snack is inexpensive, low-quality food in small portions that was packaged and unhealthy.
Wansink et al. [55]	Classification of snacks: Healthy: contains low amounts of fat, cholesterol, and sodium. Unhealthy: contains high amounts of fat, cholesterol, and sodium. Other	Among students, purchases of healthy snacks decreased over fall semester but increased over the spring semester by 4% in the final two weeks. Within semesters, unhealthy snack food choices increased significantly by about 0.4% each week. Furthermore, a sharp (8%) increase occurred in the final two weeks of the semester.
Xu et al. [65]	Classification of snacks by frequency: 0, 1, 2, 3, and ≥4 times per day.	Participants who snacked more frequently tended to be younger, consume ≥3 meals per day, and have comorbidities, a faster gait speed, and less energy from meals. Both higher snacking frequency and percentage of energy intake from snacking were positively associated with a faster gait speed.
Zizza et al. [10]	Snacks: Snack, beverage, merienda, entre comida, botana, bocadillo, tentempie, and bebida.	A total of 97.3% of participants snacked at least once during a 2-day food intake assessment, and the average number of snacks was 2.1 per day. The contribution to daily vitamin totals from snacking ranged from 9.9% for vitamin B-12 to 16.0% for vitamin E. Snacking contributed 8.8% and 4.9% of the daily intake of beta carotene and lycopene, respectively. Among minerals, the contribution to daily mineral totals from snacking ranged from 18.0% for calcium to 9.4% for selenium.

Among these studies, three tools or analytical systems appeared most commonly. The Healthy Eating Index (HEI) was used in six studies [45,46,54,58,60,64] to assess the quality of selected foods and/or general diet composition. The Dietary Guidelines for Americans was referred to in three of the studies [45,56,60]. It was used to demonstrate the contribution of snacks to meeting these guidelines, for example for eating more whole fruits [73]. Additionally, 24 h dietary recall was used in ten of these studies [3,45,46,47,54,58,60,64,65,67]. Using these tools, these studies underscored positive and negative aspects of snacks. For example, it was determined that snacks and sweets, which were considered snacks in some studies, accounted for about 20% of daily consumption of refined grains among study participants.

From the articles included in this review, three basic themes related to snacking were identified. The first was consumer cues and motivations [47,48,49,52,55,56,57,59,62,63], for example, the influence of stress on snack food choice. This was reported in one study as a shift from healthy to unhealthy snacking among university students, during the last weeks of the semester [55]. The second theme, which was more operational, had to do with snacking vs. any eating occasion [10,44,45,46,47,48,49,50,51,53,58,60,61,64,65,66,67,68,70,72]. Some studies isolated snacking as a specific or independent eating occasion; some studies considered daily food consumption as a whole, integrating the patterns and behaviors associated with snacks and meals. The third theme dealt with diet composition and health [3,10,44,46,48,53,54,58,60,61,64,66,67,68,69,71,72]. Studies highlighting snacking frequency, timing, or composition relative to diet quality and/or weight maintenance/management were included in this grouping.

## 4. Discussion

This is the first scoping review, to our knowledge, which summarizes the evidence on U.S. adult snacking, specifically. To date, only three scoping reviews have been published on the topic of snacking. One of these focused on snacking recommendations worldwide [39], another focused on socioeconomic status and snacking among adolescents [34], and one reviewed the development of snack foods for school feeding programs in Africa [73]. Only 31 studies were found to have been published between January 2010 and May 2022 with relevance to snacking among adults in the U.S. Though studies were limited in number, the evidence gleaned from these studies can further inform nutrition-related practice, research, and policy.

### 4.1. Description and Classification of Snacking Occasion

A snack is commonly defined as any eating occasion outside of a typical mealtime. This definition was promoted by nine groups of researchers [3,44,48,51,55,62], in conjunction with a stipulation from one research team that a snack is any given food item that contributes more than 50 kcal. This definition provides a framework for discussions on snacks/snacking occasions based on time of day and energy or amount of food consumed [3]. The classification related to the time of day snacking occurs is divided into daytime snacking, which includes subclassifications of morning, mid-morning, mid-day, mid-afternoon, evening [44], and late-night snacking [20].

The second classification is based on the nutrient quality of the snacks. This category differentiates between high-quality snacks, referring to individual nutrient-dense food (e.g., meat or grain/starch or fruits/vegetables); low-quality snacks, an individual nutrient-poor food (e.g., potato chips, soft drinks); and mixed-quality snacks, a combination of foods from the two other groups (e.g., an apple and some milk chocolate) [51]. Here, the quality might vary with portion size (e.g., a large apple with a small piece of chocolate vs. a small apple and a large chuck of chocolate).

The third classification involves the concept of health as applied to snacks. This dichotomous classification includes healthy snacks, which are those rated as lower in fat, cholesterol, and sodium on a per serving basis (e.g., fruit, vegetables, or low-fat yogurt); and unhealthy snacks, which are those with higher amounts of fat, cholesterol, and/or sodium per serving (e.g., pastries, fried pig skin, or frozen pizza) [55,62]. This third category shares some characteristics with the second category, but is distinguished by the exclusive focus on fat, cholesterol, and sodium. For example, a smoked-meat-based snack could be nutrient dense, but not necessarily healthy when considering fat and sodium. These three classifications are useful in assessing snack foods and snacking behaviors as a way to meet or to exceed daily nutrient and energy needs.

### 4.2. Snack Frequency and Time Periods

On average, U.S. adults snack between 1.5 and 3 times per day [10,44,48,53,62,65,70]. One study differentiated between healthy and unhealthy snacking, reporting that healthy and unhealthy snacks were consumed at a mean rate of 0.6 and 0.8 times per day, respectively [62], both below the average. Daytime snacking behaviors appear to align with the average intake, with 1 to 2 snacks/daytime period. On the other hand, late-night snacking tends to be more extreme, with up to four snacks reported among late-night snackers [56]. Estimates of snacking frequency varies somewhat according to mode of data collection. When collected and reported from energy intake estimations, the frequency was approximately 1.67 snacks/day. The same frequency (1.68) was noted with self-reports. However, when collected using a subjective tool, snacking appeared to be less frequent at 1.38 snacks/day. In general, daily eating occasions, both meals and snacks, range from three to seven [47,61,62,68]. For example, one study reported 6.5 daily eating occasions, of which 1.69 were attributable to snacks [62].

Snacking occurs throughout the 24 h day, spread over morning, evening, and night. Typically, morning snacking occurs from 8:00 to 11:30 a.m., with the first snack closer to 10 a.m. Afternoon snacking tends to occur from 1:00 to 5:30 p.m., and night snacking occurs from 8:00 p.m. to midnight, with the last snack for most people being consumed closer to 9:00 p.m. [20,44,53,68]. Among these timeframes, the morning snack is reported as the least common and the afternoon snack as the most common [53]. Two peaks of food consumption coincide with the snacking hours of 2:00 to 5:00 p.m. and 8:00 p.m. to midnight [52,58]. The average daily eating interval of both meals and snacks, from first to last bite, is 11.6 h [60], with eating occasions completed within a 15 minute timeframe. Approximately one-quarter of eating occasions occurred between 7:00 p.m. and midnight [52]. This refers, primarily, to “night snacks”; consumption of a meal (dinner) during this timeframe tends to be limited. Given the timeframe, snacks appear to provide only 20% of daily energy intake [46]. Therefore, typical peaks of consumption can be an opportunity to promote healthy snacks options, such as fruits and vegetables.

### 4.3. Perceptions and Drivers of Snacking

The act of snacking has been referred to as a personal eating event [48]. In one study, the snacking event was profiled as inexpensive, low-quality, unhealthy food that is packaged and served in small portions, consumed over a 10-min period, alone, while standing or using paper plates and napkins [71]. This is a stereotypic view of snacking. However, it aligns with the reality that snacks are frequently a grab-and-go eating experience. For example, on a university campus over two semesters, 44% of all food purchases comprised quick, convenient, unhealthy snacks, while only 22% comprised healthy snacks [55].

Notably, taste was found to be the most fundamental factor driving snack food selection [46], with the importance of other factors and predilections shifting throughout the day. Morning snacking tended to be driven by liking (taste), need, hunger, health, convenience, and weight control, resulting in a greater selection of low-calorie/low-fat satisfying foods [48]. Afternoon snacking was an interplay of liking, need, hunger, health, convenience, and weight control [48], and was further characterized by distracted eating [44]. This type of afternoon snacking was associated with individuals with higher BMI, obesogenic dietary patterns, and those less concerned with health and weight management. Finally, night snacking tended to be driven by liking, hedonics, and visual appeal with less attention to weight control [48]; therefore, night snacking is less likely to include healthier items [50]. Drivers of snack frequency appear to be related to physiological and emotional circumstances, such as hunger, need, health, and stress. In addition, environmental or social cues (site or smell) and the influence of others (social approval) are relevant drivers of afternoon snacking.

Data collected during this review also permitted us to identify three types of food purchasers. Value-loyal consumers are those who prioritize price, are brand and store-loyal, and tend to purchase foods out of habit. Emotional consumers are those who tend to be confused or overwhelmed by an abundance of choices. These purchasers can be impulsive or careless. There is also the group of high-conscious consumers, who tend to be perfectionists, thoroughly scrutinizing each of their decisions [44]. There are times, however, when a person just needs or wants food, such that any of these factors or categories becomes irrelevant [56].

### 4.4. What Foods Constitute a Snack?

Snacks can be identified as a solid or liquid food, synonymous, in Spanish, with the following expressions: botana, bocadillo, tentempie [10]. Some of the foods reported as snacks include fruits [20,44,53,62,70]; vegetables [44,53,62]; sweets; candy; sweet baked products such as cookies, pies, cakes, or pastries [20,52,57,62,69]; dairy products [62,71]; nuts; seed products; legumes and legume products; fast foods; chips; frozen dessert; deep-fried foods; and refined grain breads [20,57,62]. Liquids, such as fruit juices, milkshakes, smoothies, and carbonated and soft drinks [20,69], might also be referred to as snacks. Snacks span a diverse assortment of food products, most of which are easy to carry to work or school, readily available for purchase (kiosk or vending friendly), and easy to consume anywhere, including at home. Convenience is a common denominator for snacks.

In general, dairy products (yogurt and milk specifically) and fruits were reported as the most nutrient-dense snack categories. On the other hand, ice cream treats, pies, cakes, and carbonated drinks were reported as the most nutrient-poor snacks [69]. Snacking on sweetened baked products tend to occur in the home [52]. This makes sense because it can involve the experience of preparing and baking, as well as an opportunity to consume the product when it is still warm. Typically, sweets and fast foods are consumed at night more frequently than at other snack times [20]; nuts, seed products, fruits and vegetables are more popular as daytime snacks, though these foods are part of nighttime snacking as well [20,44,53]. Morning snacking has been associated with increased fruit and vegetable consumption [44]. However, snacking at any time of day can be a means of incorporating healthy foods into the daily diet [53].

### 4.5. Effects of Snacking on Energy and Body Weight

Several beneficial effects of snacking on health were found during this review. Males who consumed two snacks per day tended to be at a lower risk of elevated waist circumference; however, overall, snacking among males or females presented no association with weight status [3]. It was also found that higher snacking frequency and percentage of energy intake from snacking were positively associated with faster gait speed [65]. Among older adults, as snacking frequency increased, daily intake of vitamins A, C, E, B-6, beta carotene, magnesium, copper, and potassium increased [67]. Among all snackers and snacking occasions, protein intake from snacks ranged from 4.9 to 16.5 g/day [66]. In addition, snacks can contain substantial amounts of micronutrients, such as calcium, potassium, vitamin D, and magnesium [69].

It has been reported that energy consumption from daily snacks is about the same as energy consumed from an average breakfast [70]. On average, U.S. adults consume 2227 kcal/day between 8:00 a.m. and midnight [50]. This includes meals and an average of 2.8 snacks per day. Beyond routine three meals a day, an additional eating occasion or eating closer to bedtime is associated with greater caloric intake [68]. Among pregnant and postpartum women, the energy associated with each additional eating occasion was found to add 161.6 kcal/day and 146.4 kcal/day, respectively [58]. Among the general adult population, each additional eating occasion was found to add (on average) 101.8 kcals to 120 kcals/day [3]. Furthermore, among this group, those refraining from food intake within 2 hours of bedtime tended to consume 53 to 235 kcal/day less [60]. The value of snacking frequency, timing, and volume depends on individual needs.

## 5. Strengths and Limitations

The strength of this review is the characterization of a snack in the context of both a product and of an act. Evidence presented in this review helps describe the influences of time of day, environment, and social factors as drivers of adult snacking behaviors and begins to explore the impacts of snacking on energy intake and body weight.

The main limitation of this study is tied to the current literature, which does not consistently differentiate snacking from other eating occasions. Most of the 31 studies presented information (e.g., food selection, portion sizes, frequency) that included but did not necessarily isolate snacks or the snacking occasion. The data collection tools or methods that distinguished snacks from meals delivered particularly useful insights.

## 6. Conclusions

As part of the adult diet, snacks provide energy and help increase consumption of critical nutrients. The strategic selection of fruits, vegetables, and low-fat dairy as snack foods can help improve diet quality.

Snacking can be driven by internal or external cues, such as hunger, time of day, sensory perceptions, social conventions, and access. Still, snacks and snacking reflect individual decisions, which could be further influenced through nutrition education and programs.

To further advance snack-based research and health promotion, there is need for a standard, universally recognized definition of snack. This could strengthen the quality of snack-based information available to both snackers and researchers, and could further progress development of policies, nutrition programming, ultimately contributing to diet quality, health, and wellbeing.

## Figures and Tables

**Table 1:** Research strategy.

Set Number	Search Term
1	Filters: English, Spanish; Publication date from 2010 to 2022
2	(Adults Food Intake [tiab] OR “Adults Food Intake” [tiab] OR Convenience Foods Among Adults [tiab] OR “Convenience Foods Among Adults” [tiab] OR Food Acceptability Among Adults [tiab] OR “Food Acceptability Among Adults” [tiab] OR Food Purchase in Adults [tiab] OR “Food Purchase in Adults” [tiab] OR Adults Snacking [tiab] OR “Adults Snacking” [tiab] OR Snacking Frequency [tiab] OR “Snacking Frequency” [tiab])
3	(Food Intake [Mesh] OR “Food Intake” [Mesh] OR Convenience Foods [Mesh] OR “Convenience Foods” [Mesh] OR Food Acceptability [tiab] OR “Food Acceptability” [tiab] OR Food Purchase [tiab] OR “Food Purchase” [tiab] OR Snacking [Mesh] OR Snacking Frequency [tiab] OR “Snacking Frequency” [tiab])
4	(Food Intake In Adults [tiab] OR “Food Intake In Adults” [tiab] OR Adults Food Intake [tiab] OR “Adults Food Intake” [tiab] OR Convenience Foods Consumption [tiab] OR “Convenience Foods Consumption” OR Convenience Foods [Mesh] OR “Convenience Foods” [Mesh] OR Food Acceptability [tiab] OR “Food Acceptability” [tiab] OR Food Purchase [tiab] OR “Food Purchase” [tiab] OR Frequency Of Snacking [tiab] OR “Frequency Of Snacking” [tiab] OR Snacking Frequency [tiab] OR “Snacking Frequency” [tiab] OR Eating Occasions [tiab] OR “Eating Occasions” [tiab] OR Junk Food Consumption [tiab] OR “Junk Food Consumption” [tiab])
5	(“Food Intake in Adults” [tiab] OR “Adults Food Intake” [tiab] OR “Convenience Foods Consumption” [tiab] OR “Convenience Foods” [Mesh] OR “Food Acceptability” [tiab] OR “Food Purchase” [tiab] OR “Frequency of Snacking” [tiab] OR “Snacking Frequency” [tiab] OR “Eating Occasions” [tiab] OR “Junk Food Consumption” [tiab])

[tiab] = limit to title or abstract. [Mesh] = Medical Subject Headings.

## Data Availability

The data presented in this scoping review are presented in Table 2 and Table 3 of this review.
